# On Relapsing Fever

**Published:** 1870-04

**Authors:** Austin Flint

**Affiliations:** Professor of the Principles and Practice of Medicine, and of Clinical Medicine, in the Bellevue Hospital Medical College


ARTICLE I. — *On Relapsing Fever.* A Lecture by AUSTIN FLINT, M.D., Professor of the Principles and Practice of Medicine, and of Clinical Medicine, in the Bellevue Hospital Medical College.

[THE general interest felt in the profession and pervading the public mind on this subject, will cause our readers to excuse the less amount of our translations, and the space occupied by the following valuable observations by Prof. Flint. — ED.]

GENTLEMEN: Some weeks since, when I took up in order (as I have done for the last nineteen years), in my didactic course, relapsing fever, I stated that there were cases then under observation, at Bellevue Hospital, which presented the characters of that disease. I had not then observed the cases sufficiently to feel quite sure concerning the nature of the disease. I afterward stated that there could be no longer any doubt as to the correctness of the diagnosis, and promised to devote to the disease some further consideration. I propose to redeem that promise in my lecture to-day.

Let me first name the publications which you may consult for facts, chronological and geographical, relating to the past prevalence of this disease; and also, for a fair exposition of the knowledge contained in medical literature respecting it. I shall content myself with doing this, for I do not wish to occupy your time with historical details which you can, if you choose, glean from books at your leisure.

You will find this disease treated of concisely in most, if not all, of the late works on the Practice of Medicine. It is considered fully in Murchison's excellent treatise on the continued fevers of Great Britain. In the *British and Foreign Medico-Chirurgical Review*, number for July, 1851, you will find a very able article on the Continued Fevers, including relapsing fever, with numerous bibliographical references. A list of successive epidemics, in Scotland, Ireland and England, is given in that paper. Dr. William Jenner, in addition to an account of an epidemic which he studied in 1847, submits facts establishing the non-identity of relapsing with typhus and typhoid fever, in a paper published in the *Medico-Chirurgical Transactions*, vol. xxxiii, new series, 1850.

In this country, exclusive of systematic treatises on the Practice of Medicine, medical literature contains very little relating to relapsing fever. Judging from the little that has been published, the disease, with a very few exceptions, has not heretofore existed on this side of the Atlantic. Dr. Meredith Clymer observed and described fifteen cases, occurring at Philadelphia in 1844.* Dr. A. Dubois reported a few cases, occurring in New York, in 1848.† I recorded fifteen cases at the hospital in Buffalo, 1850–'51; and I gave the results of the analysis of these cases, together with an account of the disease as described by British writers, in my work entitled, "Clinical Reports on Continued Fever, based on an Analysis of one hundred and sixty-four Cases." This work, which I suppose to be now out of print, was published in 1852.

The circumstances under which these fifteen cases came under my observation were as follows: In 1850–'51, I was engaged in the study of continued fever. Of forty-eight cases which I recorded and analyzed in those years, following the example of Louis, fifteen were distinguished by the occurrence of a relapse

* *Vide* Treatise on Fevers, 1841; also, Notes in Aitken's Science and Practice of Medicine. American edition.

† *Vide* Transactions of American Medical Association, vol. ii.

after convalescense had appeared to be established. I was then unacquainted with the distinctive characters of relapsing fever; and, of these fifteen cases, nine were included in the group of cases of typhoid fever, one case was considered as typhus, and five cases were placed by themselves and called cases of doubtful type. The question, whether these fifteen cases were cases of relapsing fever, arose after reading the article in the *British and Foreign Medico-Chirurgical Review* to which reference has been made. I then analyzed these fifteen cases separately, and found that the facts corresponded, in all respects, with the history of relapsing fever. The results of the analysis are given in my "Clinical Reports on Continued Fever," and I shall refer to them, in connection with the clinical history of the disease, in this lecture.

Some of the different names by which the disease is now, and has been heretofore known, are to be mentioned. In past literature it has been called "five-day fever," "seven-day fever," "short fever," "mild yellow fever." By some of the British writers it is distinguished as "famine fever," and by some German authors as "hunger pest." The name "relapsing fever" is based on the most striking of its peculiarities, and it involves no hypothesis; it is therefore to be preferred to any other, with our present knowledge.

By way of introduction to the consideration of important questions concerning the disease, I will read a report of one of the first of the cases received into the hospital — a case which is typical as regards the prominent, distinctive features of this species of fever; and I will then introduce a patient, who is supposed to be passing through the primary paroxysm, and also a patient who has passed through the disease, and is now convalescing. The report which I shall proceed to read was drawn up by Dr. William M. Polk, senior assistant physician on the third medical division:

Henry Gordon, aged seventeen, a native of this country, by occupation a laborer, was admitted December 17, 1869. He came from No. 37 Mulberry street, and he stated that several persons in the house in which he lived had been ill with the same kind of fever. He had been exposed to many hardships, inclusive, probably, of a want of proper food.

He was well, up to four days before his admission, when he was seized with a chill, followed by fever and pain in the limbs. He had no epistaxis, nor diarrhœa. He suffered much from headache. On his admission the pulse was 110. He then complained of severe muscular pains. The bowels were constipated. The skin was hot, but moist. There was no eruption; there was no jaundice.

December 18, the day after his admission, he was free from fever. The pulse and temperature were normal. There was profuse perspiration on this date.

He continued free from fever from the 18th to the 28th, that is, ten days. The last four of these ten days he was sitting up. He continued, however, to complain of muscular pains, and he was quite weak.

On the 28th of December, after getting up, at 7 A.M., he felt chilly sensations, and soon returned to bed. The pulse was now 100, and the temperature in the axilla was 100°. At evening the pulse was 110, and the temperature 101°. On the 29th, the pulse was 120 at A.M., and the temperature 102°. He had epistaxis for a few moments, on this date, and diarrhœa. The fever persisted, without any abatement, for the two following days, the pulse being 120, and the temperature 102°. On January 1, at 9 A.M., the pulse fell to 90, and the temperature to 100°. He was then perspiring profusely. At 6 P.M. the pulse and temperature were normal. Free perspiration continued. The muscular pains had continued to be, and were still, severe.

The relapse of fever thus ended, after a continuance of five days. After January 1 there was no return of fever. On the 13th of January he was able to sit up. He convalesced without any drawback, and was disharged, quite well, January 24. There was no eruption of any kind. The diarrhœa continued only two days, and was slight.

The treatment was as follows: Ten grains of quinine were given daily, for the first four days, in one dose, at noon; and two grains three times daily, during the intermission. Dilute nitric acid was given during the continuance of fever. Bismuth, kino, and opium, were given to relieve the diarrhœa.

The points in this case to which your attention is to be directed are, the abruptness of the attack; the continuance of high febrile movement for five days; the sudden and complete disappearance of fever, profuse perspiration occurring at the same time; an apyrexial period of ten days; an abrupt relapse of fever, lasting five days, the second febrile career ending suddenly, with profuse perspiration, and convalescence then taking place; the prominence of muscular pains, both during the fever and in the intermission; the residence of the patient in a house where this fever existed; his suffering from deprivations before being attacked; and, finally, the absence in the history of the characters which distinguish, severally, typhus, typhoid, and remittent fever.

I shall now introduce a patient who is supposed to be passing through the primary febrile career of relapsing fever. The patient is a girl, fourteen years of age. She was admitted with fever, which has now continued for five days. The pulse is 140, and the axillary temperature 101°. Now let us consider the question, What are the grounds for supposing this to be a case of relapsing fever?

In the first place, it is an essential fever of some kind, for a careful examination of the different organs of the body has failed to disclose an acute inflammation anywhere; it is not, therefore, a symptomatic fever. It is not one of the eruptive fevers, for there has been ample time for the eruption and other diagnostic characters of these fevers to become manifested, and they are wanting. If not relapsing fever, it must be a febricula, or remittent fever, or typhus, or typhoid fever. The duration and the intensity of the febrile phenomena show that it is not a febricula. None of the characters of remittent fever have been exhibited. The abruptness of the attack, and the absence of the abdominal symptoms of typhoid fever, warrant us in saying that it is not, in all probability, that disease. Finally, were the disease typhus fever, it is time for the eruption to have appeared, and this is rarely wanting in cases of typhus. The eruption is not present in this case; moreover, there is not the dusky hue of the face which characterizes typhus; nor is there the physiognomy representing the mental condition which belongs both to typhus and typhoid fever. There has been no incoherency or passive delirium, and the patient is now able to give an accurate account of the past and present symptoms.

Thus, gentlemen, you see that the diagnosis is reached, at the present time, in this case, chiefly by exclusion; but, if the disease be relapsing fever, we may expect daily a sudden cessation of the febrile symptoms, and a period of apyrexia, or an intermission, which will probably be followed by a relapse. The diagnosis will then be confirmed.*

Before this patient is removed, let me call your attention to the presence of petechiæ on the lower limbs. The small, round, dark spots which you see are truly petechial, that is, they are minute extravasations of blood, or ecchymoses. They may occur in relapsing fever, as in typhus or typhoid fever, and also, in various diseases. They have no diagnostic significance, nor do they denote an unusual gravity of disease.

I now introduce a patient who is convalescing from relapsing fever. The first career of fever was seven days, in this case; then followed an intermission of seven days. The relapse of fever lasted only four days. He is able to walk up to the amphitheatre, and is nearly well enough to be discharged.

The remainder of my lecture I shall devote to a brief consideration of the following questions: What points in the clinical

* The subsequent history of this case corroborated the diagnosis.

history of relapsing fever are distinctive of the disease; and has it any anatomical characteristics? What are the grounds for considering this disease as a distinct species of fever? What are the points involved in the diagnosis of relapsing fever? What is the existing state of our knowledge of its causation? What is the prognosis? And, lastly, what are the indications for treatment? These questions I shall take up *seriatim*, following the order in which they are stated.

*What points in the clinical history of relapsing fever are distinctive of the disease; and has it any anatomical characteristics?* My remarks under this head, as well as in relation to the other questions, will be based, not exclusively on the information to be obtained from the writings to which reference has been made, but on the observations in this hospital during the present prevalence of the disease. Through the zeal and assiduity of the house physician of the third medical division, Dr. Thomas J. Moore, I have been furnished with sixteen recorded histories. These histories have been recorded with care, the pulse and axillary temperature having generally been noted twice daily. I am much indebted to this gentleman for his interest and fidelity. I should add that most of the cases were under my observation. Dr. Moore has also furnished a tabulated statement of statistical facts relating to the cases throughout the hospital.* I have carefully analyzed the sixteen cases just referred to. In addition, I have the results of an analysis of the fifteen cases which I observed and recorded in 1850–'51. The two collections thus make thirty-one recorded cases.

Abruptness of invasion characterizes the disease. The attack is sudden. There is no prodromic period. The seizure is almost always marked by a well-pronounced chill, which is immediately followed by febrile movement. Usually, the patient at once takes to the bed; but, in some cases, one, two, or three days pass before there is this evidence of yielding to the disease. Moderate perspiration occurs shortly after the fever begins, in a considerable proportion of cases. This was noted in seven of twelve cases which I formerly observed. It is noted in the histories of several of the cases recently under observation. The perspiration in some cases is abundant, and it may recur repeatedly during the continuance of the febrile paroxysm.

The fever attains quickly to either considerable or great intensity, as denoted by the pulse and axillary temperature. Thus, of two cases in which the disease was developed in the hospital, in one, the pulse on the first day was 120, and the temperature 103°; in the other case, the pulse on the first day was 130, and the tem-

* This statement is appended to the lecture.

perature was 103°. During the continuance of the first paroxysm, the pulse and temperature generally denote a persistent intensity of fever, the pulse ranging, in different cases, from 100 to 140, and the temperature from 100° to 105°. The oscillations are rarely great, and those which occur are irregular in their occurrence.

The cessation of the fever is as abrupt as the accession. The pulse and temperature quickly fall to nearly or quite the normal standard. The transition from high fever to complete apyrexia takes place, often, in a few hours; usually this is accompanied by profuse perspiration, which continues for several hours, and even an entire day. Not infrequently, the pulse and temperature fall below the standard of health. In two of the cases recently under observation, the pulse fell to 54, and in one case the temperature to 95°. In a day or two both the pulse and temperature rise again to the normal standard.

The duration of the primary paroxysm is stated to be, in the majority of cases, from five to seven days. Exceptionally, it may be only two days, and it may be twelve days. In the cases which I formerly observed, the average duration was nine days, the maximum being twelve and the minimum six days. Of ten cases, among those recently observed, in which the duration was definitely determined, the minimum was four days, the maximum eight days, and the average duration was a fraction under six days.

During the apyrexial period or intermission, the absence of fever is complete. It is incorrect to call this period a remission; the fever does not remit, but it intermits. The average duration of this period is stated to be about seven days; but it may not exceed two or three days, and it may extend to twelve days, or even more. In the cases which I formerly observed, the average duration was five days, the longest being eight, and the shortest three days. Of the cases recently observed, the minimum duration of this period was five days, and the maximum nine days. The mean duration was a fraction over six days.

The relapse, like the primary attack, is sudden. It is generally ushered in by chilly sensations, but not so constantly by a well-pronounced chill as the first paroxysm. The fever in the relapse quickly becomes more or less intense. The intensity may exceed that of the first paroxysm. But, in the majority of cases, the intensity is less. The relapse also ends suddenly, and in most cases with profuse perspiration.

The duration of the relapse is stated to vary between three and five days. It may, however, be only twenty-four hours, and it may extend to ten days. In the cases which I formerly observed, the minimum was two days, and the maximum ten days. The mean duration was six days. Of the cases recently under observation, the duration of the relapse is noted in eleven. The shortest duration was three days, and the longest was eight days. The average was four and a half days.

It is to be borne in mind that the relapse does not always take place. It was apparently wanting in one of the cases recently observed. On the other hand, a second, a third, and even a fourth and a fifth relapse have been observed. In none of the cases which I have seen has there been more than a single relapse.

To illustrate the course of the disease, as represented by the pulse and axillary temperature, I will read the daily record of these symptoms in the history of a case which is selected as typical.

The patient was admitted into Bellevue Hospital on the fifth day of the disease:

| | PULSE. | TEMPERATURE. | |
|---|---|---|---|
| 5th day | 105 | 102.5° | |
| 6th " | 104 A.M. | 103.5° A.M. | |
| " " | 108 P.M. | 103.5° P.M. | |
| 7th " | 90 A.M. | 102.5° A.M. | |
| " " | 96 P.M. | 101° P. M. | |
| 8th " | 88 A.M. | 103° A.M. | Profuse |
| " " | 114 P.M. | 104° A.M. | perspiration. |
| 9th " | 60 A.M. | 96° A.M. | |
| " " | 68 P.M. | 97° P.M. | |
| 10th " | 60 A.M. | 98° A.M. | |
| " " | 54 P.M. | 96° P.M. | |
| 11th " | 56 | 98.5° | |
| 12th " | 64 | 98° | |
| 13th " | 64 | 98.5° | |
| 14th " | 100 A.M. | 105° A.M. | |
| " " | 105 P.M. | 105° P.M. | |
| 15th " | 96 | 102° | |
| 16th " | 114 | 104° | Profuse perspiration. |
| 17th " | 66 | 91° | |

The points which have been presented are those most highly distinctive of relapsing fever. It remains to notice certain other points, belonging to the clinical history, which are more or less characteristic.

Of symptoms referable to the digestive system, nausea and vomiting occur sufficiently often to be somewhat distinctive, especially when this disease is contrasted with typhus and typhoid fever. Not infrequently, these symptoms are prominent and persistent during the febrile paroxysms. The matter vomited is green or yellow, from the presence of bile. The tongue presents nothing distinctive; it is generally coated, and in some cases becomes dry and fissured. The vomiting of blood, presenting the character of "black vomit," has been observed; but it is a very rare symptom, and I have not heard of its occurrence in any case during the present prevalence of the disease. It is generally associated with hæmorrhage in other situations, and it is to be considered as an accidental event, not as an element of the disease. It probably denotes a scorbutic complication. Diarrhœa occurs very infrequently; constipation is the rule. The diarrhœa, when it occurs, is evidently accidental. Meteorism, in a moderate degree, is not uncommon; it existed in nine of the fifteen cases which I formerly observed. In only one case, however, was there any considerable tympanitic distension of the abdomen. Tenderness, on pressure, over the epigastric region, existed in six of these fifteen cases. Slight tenderness in the iliac region is not uncommon. Notable tenderness exists, in some cases, over the liver and spleen; and enlargement of these organs is sometimes determinable by palpation and percussion. I have not observed a craving for food during the paroxysms, which, according to some writers, is distinctive of this form of fever. The appetite, however, returns during the intermission, and the digestion may be active in this stage.

The occurrence of jaundice may be mentioned in this connection. This event occurs in a small proportion of cases, but its infrequency in the other continued fevers renders it somewhat distinctive of relapsing fever. It was present in two of the fifteen cases which I formerly observed, and in four of the sixteen cases recently under observation. In these six of thirty-one cases the jaundice was slight, and the cases in which it was present were not unusually severe. It is doubtful if the statement that this event is an element of gravity be correct. The event is much more frequent in some epidemics than in others. The name "mild yellow fever," which has heretofore been applied to some epidemics of relapsing fever, derives whatever pertinency it has, from the occurrence of jaundice, sometimes in a considerable proportion of cases, and also from the occasional occurrence of black vomit.

A symptom referable to the nervous and the muscular system, is highly distinctive of the disease under consideration. I refer to arthritic and muscular pains, more especially the latter. During the first paroxysm, pains in the loins, the calves of the legs, and the muscles in other situations, are generally much complained of. They are never wanting, although, as regards intensity, they differ considerably in different cases. The muscular pains do not cease with the ending of the paroxysm, but they continue during the intermission; they are more or less prominent during the relapse, and they are apt to persist into convalescence.

The mental condition, perhaps, in a measure, accounts for the suffering from these pains. The perceptions are not blunted in this disease as they are in typhus and typhoid fever. This is a negative point of distinction in contrast with the fevers just named. Another negative point is the absence of the delirium which characterizes typhus and typhoid fever. Delirium is by no means absent in all cases of relapsing fever; but the delirium is such as is apt to occur whenever there is high febrile movement, whether the fever be essential or symptomatic, and it is generally manifested only at night. In the daytime the mental faculties are generally intact. The condition known as coma-vigil does not belong to the clinical history of relapsing fever. This statement is also true of subsultus, carphologia, and other ataxic symptoms which occur in grave cases of typhus and typhoid fever. Deafness is also a rare symptom in relapsing fever.

A distinctive point, in comparison with typhus and typhoid fever, is the absence of a characteristic eruption. In most cases there is no eruption. Sudamina or miliary vesicles are sometimes observed at the time when profuse perspiration occurs; but this eruption is incidental to various affections. The same is true of petechial spots which occur in some cases of relapsing fever. Other kinds of eruption are sometimes accidentally associated. Even the rose papules of typhoid fever sometimes occur in this as they do in various other diseases.

The physiognomy presents nothing distinctive. The face is more or less flushed, as in cases of symptomatic fever. There is not that degree of capillary congestion, marked especially on the cheeks, which exists in typhoid fever, nor the dingy complexion which characterizes typhus. The expression of indifference, vacuity, or stupidity, which is a notable characteristic of the fevers just named, is rarely observed in relapsing fever. In one of the cases recorded by Dr. Moore, it is stated that, in conjunction with slight jaundice, "the face was flushed, as if there existed erysipelas, excepting the cheeks, the latter being quite pale, and the contrast giving a very peculiar appearance."

The urine in relapsing fever is yet to be studied fully. It appears, however, that, as a rule, the quantity of urine is increased, and the urea is in larger proportion than in health. Great diminution, and even suppression of the urine, however, are sometimes observed, uræmic coma and convulsions taking place, but these cases are, happily, exceedingly rare.

It may be stated, as a point somewhat distinctive of relapsing fever, that there is very little liability to serious complications. In this fact we have an explanation, in part at least, of the very small rate of fatality from the disease. Pneumonia, however occurred in three of the fifteen cases which I formerly observed; but this complication, in these cases, did not prove fatal. It did not occur in any of the cases recently under observation. Of the latter cases, in three, mild bronchitis was a complication. Diarrhœa and dysentery have been observed to occur not infrequently in some epidemics.

What has just been stated with respect to complications holds true as regards sequels. As a rule, important sequels do not occur. A peculiar form of ophthalmia, which was described as following the cases reported by Dr. A. Dubois, of this city, in 1848, is an exception to the rule. It has been repeatedly observed as a sequel of relapsing fever. The peculiarities of the ophthalmia are described in works which treat of the diseases of the eye. This sequel, however, has not occurred in any of the cases which I have observed. Relapsing fever, when it attacks pregnant women, almost always leads to miscarriage or abortion. The mother almost invariably recovers, but the child, no matter how near may be the end of gestation, as a rule, is either stillborn, or dies shortly after birth.

There are no constant lesions found after death which are distinctive of this disease. The spleen is uniformly more or less enlarged and softened; but this occurs in typhus and typhoid fever. The liver is, also, more or less enlarged, but without any special appearance or structural change. Changes in other organs, which may be found, are due either to complications or to antecedent disease. It is a negative point of distinction in contrast with malarial fevers and with typhoid fever, that relapsing fever is devoid of any anatomical characteristics.

*What are the grounds for considering this disease as a distinct species of fever?* Is relapsing fever a special form of disease; in other words, is it a distinct species of fever? I think facts warrant a positive answer to this question in the affirmative. Let us consider briefly the grounds for this opinion:

In the first place, the laws of relapsing fever, as regards the primary paroxysm, the intermission and the relapse, or relapses, are very striking and peculiar. The clinical history, in these respects, has but a remote analogy to the different types of intermittent fever, and to the very rare instanees in which a relapse of either typhus or typhoid fever is observed. In respect of the distinctive points now referred to, relapsing fever differs essentially from any other form of fever; it stands alone. Moreover, as we have just seen, there are other distinctive points in its clinical history. That it is a distinct species of fever is a fair inference from the peculiarities pertaining its phenomena and laws.

In the second place, if it be not a distinct disease, it must be a variety of periodical, that is, malarial fever, or of either typhus or typhoid fever. Now, it may be clearly shown to lack certain characters which are essential to the fevers just named. To prove that it is not a form of periodical or malarial fever, it is sufficient to say that it has prevailed repeatedly in situations where the special cause of intermittent and remittent fever, that is, malaria, does not exist; that it is undoubtedly a contagious disease, and that it is not controlled by anti-periodic remedies. The proof involved in these facts is so conclusive that it is unnecessary to cite further evidence. That it is not a variety of typhoid fever is shown conclusively by the absence of the anatomical characteristics of the latter disease, the so-called typhoid lesions of the small intestine; and also by the absence of the essential features pertaining to the clinical history of typhoid fever. The grounds for the non-identity of relapsing fever and typhoid fever are certainly stronger than for the non-identity of typhoid and typhus fever. The essential points of difference, indeed, are more strongly marked than those which distinguished measles from scarlet fever —diseases which were once considered as identical.

To establish the opinion that relapsing fever is a distinct disease, it is then only necessary to show that it is not a variety of typhus. It is certainly a distinct species of fever, if its non-identity with typhus be proven. The points of difference in the clinical history of relapsing fever and typhus are very marked. A relapse of typhus is exceeding rare; and, when it occurs, the duration of the primary career of fever exceeds the average duration of the first paroxysm of relapsing fever. On the other hand, the occurrence of a relapse in relapsing fever is the rule, the exceptions to which are as rare as is the occurrence of a relapse of typhus. The characteristic eruption which is nearly constant in typhus never occurs in relapsing fever, The physiognomy and the mental condition in the two diseases present wide points of difference. The fatality, which is considerable in typhus, is comparatively insignificant in relapsing fever. But the conclusive proof is that relapsing fever affords no protection against typhus. Patients who have passed through the former have repeatedly contracted, by contagion, the latter. Murchison cites an abundance of facts exemplifying the correctness of this statement. Hence it is wrong to transfer patients affected with relapsing fever to hospital wards containing cases of typhus. There are no facts showing that the contagion of typhus ever gives rise to relapsing fever. The conclusion is, that each of these two diseases has its own special poison or miasm, by means of which it alone is reproduced; that is, neither of these two diseases is capable of communicating the other. This fact is sufficient to establish the opinion of their non-identity. A fact which is distinctive of relapsing fever, as contrasted with typhus and typhoid fever, is that its having been once experinced does not afford exemption from subsequent attacks. It has been known to attack repeatedly the same person.

*What are the points involved in the diagnosis of relapsing fever?* The laws of relapsing fever, relating to the primary paroxysm, the intermission and the relapse, are so distinctive that there can hardly be room for doubt concerning the diagnosis after the disease has ended. There is difficulty in discriminating it from other essential fevers chiefly in the primary paroxysm. The differential points in this stage I have already presented in connection with the case introduced in the early part of this lecture. I shall content myself with a recapitulation of these points.

The diagnosis involves certain distinctive characters which belong to the primary paroxysm ; but it is to be based more especially, on negative points ; in other words, on reasoning by way of exclusion. The distinctive characters are the abruptness of the invasion, the rapid increment of fever, the frequent occurrence of moisture on the skin, or perspiration more or less abundant, without any marked abatement of the fever, the prominence of muscular and arthritic pains, and, in certain cases, the occurrence of jaundice. These are the positive points which are diagnostic of relapsing fever. The fevers to be excluded are the eruptive fevers, febricula, remittent fever, typhus and typhoid fever. Scarlet fever and measles are readily excluded by the absence of the eruption and of the characters which are distinctive of these fevers during the period of invasion. Small-pox is excluded after the third day by the continuance of fever and the absence of the eruption. It is not always practicable to decide at once that the disease is not a febricula ; but the intensity of the fever and the notable muscular pains do not belong to the latter. Doubt, however, is soon removed by the persistence of the fever. Typhoid fever is excluded by the absence of a prolonged access, and the absence of the abdominal symptoms, before sufficient time has elapsed for the appearance of the eruption, and of the characteristic mental condition. Typhus is excluded by the absence of the eruption, which appears earlier than in typhoid fever, by the absence of the dusky complexion, and by the absence of the mental condition—the latter also being apparent earlier than in typhoid fever. Remittent fever is excluded by the absence of remissions, these being determinable by a notable diminution of the axillary temperature in some cases in which they are not distinctly denoted by the pulse. Of course, the prevalence of relapsing fever is taken into account in arriving at the diagnosis.

*What is the existing state of our knowledge as regards the causation of relapsing fever?* Is relapsing fever communicable from the sick to the well ? Undoubtedly this question is to be answered affirmatively. This opinion rests on facts derived from the different sources of evidence, exclusive of inoculation, which establish the contagiousness of other diseases ; for example, typhus fever. Of those who are attacked during the prevalence of the disease, a large proportion are known to have been brought into contact with, or close proximity to, patients affected with it. The disease is diffused in hospitals among fellow-patients and those who have charge of the sick. During the period in which cases were received in Bellevue Hospital, after the disease began to prevail recently in this city, namely, between November 14, 1869, and February 6, 1870, twelve persons contracted the fever in the hospital. These twelve persons were especially brought into contact with patients affected with the disease, and in no instance did it attack one who had not been thus exposed. One of the senior assistant physicians residing in the hospital has had it. The orderly in one of my wards contracted it; and his wife, who came to nurse him, was attacked by it. The disease has often been diffused in localities in which it did not previously exist, after the importation of a case.

Facts, however, go to show that it is not a highly-contagious disease. Considerable exposure appears, in general, to be necessary. The area of the infecting distance appears to be limited, and it remains to be ascertained whether it may be transported by fomites. Some facts, cited by Murchison, render it probable that it may be diffused in the latter mode. It is especially communicable when the miasm is derived from a number of patients in the wards of hospital, or in close apartments. The disease is not likely to be contracted from single patients in well-ventilated rooms. The investigations of the Metropolitan Board of Health in this city, during the present prevalence of the disease, have shown that it is diffused chiefly among those living in overcrowded, illy-ventilated tenement houses. Of the propriety and importance of removing these patients to hospitals or wards devoted specially to cases of this disease, there can be no doubt.

Destitution, deprivations, and especially deficient alimentation, are powerful predisposing causes. Of this fact, the past history of the disease, at different times and places, furnishes abundant evidence. The significance of the names "famine fever" and "hunger pest" relates to this fact. It is a question whether the special poison may not be generated by the want of food and other sanitary deficiencies, irrespective of its production as an infectious miasm. I am not prepared to offer an opinion respecting this question. It is to be remarked, however, that in typhoid fever we have an example of a fever which is undoubtedly, under certain circumstances, communicable, but which, it is probable, in the majority of cases, originates independently of contagion. For interesting and important details respecting the causation, I refer you especially to the treatise by Murchison.

Statistics do not show any notable etiological influence pertaining to age, sex, or season. As regards age and season, relapsing fever thus differs from typhoid fever.

The causation of relapsing fever is of importance with reference to the inquiry whether the disease is likely to prevail indefinitely in this city, and in other parts of our country. Although its diffusion involves generally, and perhaps always, a contagious principle, the history of the disease in other countries shows that it occurs as an epidemic, and, after continuing for a certain time, it disappears completely. Thus, in the city of London, for the fourteen years preceding the winter of 1868–'69, there had been no cases of the disease. The complete disappearance of the disease for so long a period goes to show the operation of predisposing and co-operating causes in conjunction with the special poison on which the production of the disease depends. In view of the past history of the disease, it is a fair conclusion that it will not become a permanent visitor on this side of the Atlantic. It will prevail in this city as long as auxiliary causes favor its diffusion. It will be likely to be carried to other places, especially to large towns where a considerable portion of the population are living under circumstances favorable to its occurrence; but it may be expected to disappear not long after it ceases to prevail as an epidemic. The effective means for arresting the spread of the disease are, thinning the population of overcrowded tenement-houses, dispersing the occupants of insalubrious dwellings, relieving destitution, especially as regards food, and promptly removing patients to hospitals devoted to cases of relapsing fever.

*The prognosis in relapsing fever.* It is, at first view, remarkable that a fever of such intensity, and prevailing especially among a class of persons whose powers of tolerance are impaired by previous hardships and want, should have such a small rate of mortality. The mortality, in different collections of cases, varies from two to four per cent. Dr. Moore, in his statistical report, states that, of one hundred and three cases admitted into Bellevue Hospital between November 14, 1869, and February 6, 1870, only two proved fatal.* What is the explanation of this low rate of mortality? An explanation, which perhaps is sufficient to account for the fact, has been already given, namely, the disease is very rarely accompanied by any serious complications. The comparatively much greater fatality of typhus and typhoid fever is due mainly to complications. These fevers rarely kill *per se*, that is, death is not purely an effect of the intensity of the disease.

Of the fatal cases of relapsing fever, the death, in a certain proportion, is attributable either to complications, such as pneumonia and dysentery, or to antecedent affections, for example, disease of the kidneys. But this fever may destroy life, irrespective of any important complications or antecedent affection. Several observers have reported cases in which sudden death occurred appar-

* *Vide* Dr. Moore's report appended to this lecture.

ently from syncope. One of the fatal cases in this hospital apparently exemplified this fact. The death was sudden and unexpected during the night, on the seventh day of the disease. At the time of death, the primary paroxysm seemed about to end, free perspiration having taken place. Neither coma nor convulsions occurred, and, as far as information could be obtained, the dying was by syncope. The autopsy revealed no important lesions except that the kidneys appeared to be fatty.

Suppression of urine followed by uræmic coma and convulsions is sometimes a cause of death. This fact was exemplified by one of the two fatal cases occurring in this hospital. It remains to be determined whether the suppression of urine may result from simply functional inactivity of the kidneys, or whether in all cases disease of these organs exists, as either as intercurrent, or an antecedent affection.

*What are the indications for the treatment of relapsing fever?* There are no known means which can be relied upon for cutting short a relapsing fever. With our present knowledge, there are no remedies which, employed in the intermission, will prevent the relapse. Quinia has been used freely in this stage, recently at this hospital, but with no success. Its inutility, as a prophylactic, has been abundantly shown by different observers. Reasoning by analogy, I am led to think that the mineral acids may exert somewhat of that modifying influence, in this disease, which is so manifest in typhus and typhoid fever. They have been, to some extent, used in the cases recently treated here, but not sufficiently to warrant any conclusions based on experience. They certainly deserve to be tried. The treatment, with our present knowledge, must be expectant, meaning by this term, that it is to consist of palliative measures, and those addressed to the particular indications in individual cases.

The intensity of the fever during the paroxysms may be lessened by sponging the body with water, and perhaps by the wet sheet. Cold water may be taken into the stomach freely. Ice-cold carbonic-acid water is an acceptable and useful drink to allay thirst. Cephalalgia may be relieved by cold applications to the head. The bowels should be kept open by saline laxatives. The muscular and arthritic pains call for the use of opium, especially if it produce no unpleasant after-effects. Irrespective of the pains, opiates are indicated to relieve sleeplessness.

The dietetic treatment is important, more especially in the cases in which deficient alimentation has been a predisposing cause of the disease. In this fever, as in other fevers, when alimentary support is indicated, milk is the form of diet to be preferred. And it is to be borne in mind that in this, as in most diseases, there is never any danger of the over-appropriation of nutriment; the only risk is in the ingestion of more food than can be digested.

It is desirable that, during the paroxysms, from one to two quarts of milk should be taken daily. In the intermission, when the appetite returns, as much substantial food as can be digested should be allowed; and the more food is appropriated in this stage, the better is the patient enabled to tolerate the relapse. Tonic remedies are indicated throughout the disease, and especially in the intermission. Quinia in small doses, and some preparation of iron, are the tonics to be preferred.

If the symptoms denote asthenia, alcoholic stimulants are indicated. The occasional occurrence of death from syncope renders it important to watch carefully for this indication. When there is any room for supposing that alcoholic stimulants may be useful, they should be given tentatively and continued, or not, according to the effect, especially on the pulse and temperature. They are, of course, urgently indicated if the symptoms denote danger in the direction of asthenia.

Attention to the urine is important. It is to be recollected that a cause of death is suppression of urine. If the urinary excretion be deficient in quantity, or, if the quantity being sufficient, there be a deficiency of urea, diuretics are indicated. Should the kidneys not respond to diuretics, hydragogue laxatives or cathartics are indicated, with a view to eliminate urea through the alimentary canal. Active hydragogues are, of course, indicated, if uræmic coma or convulsions should supervene.

It is stated that convalescence from relapsing fever is notably slow, patients remaining for a long time much enfeebled, and tolerating with difficulty affections which may occur before the recovery is complete. This has not been a marked peculiarity in the cases which have come under my observation. It is doubtless more likely to be marked in the cases in which there had been impairment of the vital powers from innutrition and hardships before the fever was contracted; and also, it is more likely to be marked where abundant alimentation and the judicious use of stimulants have not entered into the treatment of the disease.—*New York Medical Journal*, *March*, 1870.

